# Environmental Impact of Online Versus in-Person Critical Care Education Through the Carbon Footprint Analysis of the CERTAIN Program: Cross-Sectional Study

**DOI:** 10.2196/63524

**Published:** 2025-07-31

**Authors:** Baiyong Wang, Claudia Castillo Zambrano, Nasrin Nikravangolsefid, Ricardo Machado Carvalhais, Alexander Niven, Ognjen Gajic, Yue Dong

**Affiliations:** 1 Department of Critical Care Medicine, the Affiliated Hospital of Hangzhou Normal University, Hangzhou, China; 2 Division of Pulmonary and Critical Care Medicine, Department of Medicine, Mayo Clinic, Rochester, MN, United States; 3 Department of Internal Medicine, Montefiore New Rochelle Hospital, New Rochelle, NY, United States; 4 Department of Anesthesiology and Perioperative Medicine, Mayo Clinic, 1216 2nd St SW, Rochester, MN, 55092, United States, 1 5073228432

**Keywords:** carbon footprint, CO2e emissions, continuing medical education, climate change, public health, medical education, environment, global health, digital education, eLearning, carbon dioxide equivalent emissions

## Abstract

**Background:**

Climate change is a pressing public health issue, with the US health care sector contributing about 479 million tons of carbon dioxide (CO_2_) annually. Online continuing medical education offers an alternative solution to increase global education delivery while reducing CO_2_ emissions associated with traditional teaching methods.

**Objective:**

This study aimed to evaluate the carbon dioxide equivalent (CO_2_e) emissions associated with different delivery methods of the CERTAIN (Checklist for Early Recognition and Treatment of Acute Illness and Injury) global critical care education program. Specifically, we aimed to compare the climate impact of local in-person courses in Rochester, MN, international in-person courses, and online courses to determine the potential environmental benefits of transitioning to digital education platforms.

**Methods:**

A cross-sectional analysis of CO_2_e emissions linked to the CERTAIN program was conducted from 2016 to 2022. We compared the climate impact of 3 different course offerings: local in-person at Rochester, MN, international in-person courses, and online courses. The international conferences were conducted in the host country with faculty traveling there to provide the educational content. CO_2_e emissions were calculated using the “My Climate Flight Calculator” and “Environmental Protection Agency Emission Factors” formulas for travel, conference venues, and online course–related emissions. Learner satisfaction was assessed via validated 5-point Likert surveys.

**Results:**

Local courses had the highest emissions: 52.7 tons/course (2.5 tons/participant), 96% from air travel (*50.6tons, P*<.001), versus other formats. International courses showed 20.2 tons/course (0.4 tons/participant), of which 93%(18.8 tons) were travel-related. Online courses reduced emissions by 96% per capita (0.1 tons/participant, *P*<.001) and 89% per course (5.6 tons, *P*<.001) versus local format. Overall course ratings were either excellent (live 50%, n=136) vs online 44%, n=11) or very good (live 30.9%, n=84 vs online 53%, n=12) for both live and online courses.

**Conclusions:**

The transition to online delivery of our CERTAIN global education program has led to a substantial reduction in CO_2_ emissions, mainly by eliminating travel, with similar levels of learner satisfaction. These findings support a strategic shift toward digital medical education platforms to promote environmental responsibility and broaden global educational access.

## Introduction

Climate change has been recognized as the leading threat to public health around the globe [1,2]. It is well established that rising levels of greenhouse gas (GHG) emissions cause and exacerbate a wide range of health problems due to air pollution, severe weather, wildfires, extreme temperatures, changes in vector ecology, and disturbances in the food supply, among others [3]. Health care systems are large contributors to global emissions, and intensive care units (ICUs) are a complex and resource-intensive component of these systems [4]. The US health care industry emits an estimated 479 million tons of carbon dioxide equivalent emissions (CO_2_e) each year; nearly 8% of the country’s total [5]. These emissions stem directly from health care facility operations, and indirectly from purchased sources of energy, heating, and cooling, and health care services and goods supply chain [3]. If the global health care sector were a country, it would be the world’s fifth-largest emitter of GHGs [6]. Agency for Healthcare Research and Quality identifies the climate crisis as the 21st century’s top public health challenge, urging the health care sector to urgently reduce its carbon footprint by the administration’s goals to halve emissions in 8 years and reach net zero by 2050, highlighting the necessity for medical professionals to quickly address their industry’s environmental impact [7,8].

Higher education institutions are leading in sustainability by reporting GHG emissions through the American College and University Presidents Climate Commitment, which requires them to account for their emissions and create a climate action plan with reduction goals [9]. Online medical education reduces travel and energy usage at physical venues, which benefits the environment [10]. The expansion and adoption of online learning during the COVID-19 pandemic offers a more sustainable alternative to traditional, classroom-based education [11-13].

CERTAIN (Checklist for Early Recognition and Treatment of Acute Illness and Injury) is a global critical care training program focused on systematic evaluation and continuous management of critically ill patients [14-16]. Its pedagogical structure is tailored for global dissemination, including online simulation–based activities and weekly sessions, facilitated by a diverse panel of local and international experts [17]. We aimed to compare and quantify the carbon emissions associated with our local and international in-person courses, which took place prior to the COVID-19 pandemic, with our online offering that we developed and implemented over the past 4 years.

## Methods

### Recruitment

The CERTAIN program started in 2013 as an online global quality improvement project with demonstrated improved care process and patient outcomes from 34 ICUs from 15 countries [14]. The CERTAIN education programs (2 d live workshop) were conducted in Rochester, MN from 2016 to 2018. From 2019, this program was adapted to an international in-person offering, which was offered 6 times in 5 different countries (India, Croatia, Slovenia, China, and Vietnam). In 2020, due to the COVID-19 pandemic, the program was converted to an exclusively online offering, including both asynchronous learning using a flipped classroom model and synchronous virtual sessions that included critical care topic updates, case-based discussions, simulation-based and quality improvement training [18]. We analyzed the CO_2_e associated with these 3-course delivery formats using the data of faculty and learner engagement between 2016 and 2022 recorded in our database, including (1) 24 faculty and 57 learners in Rochester, MN, (2) 26 faculty and 290 learners in our international, in-person courses, and (3) 12 faculty and 282 learners in our online offerings (Table 1).

**Table 1.** Comparative characteristics of 3 instructional modalities in the Checklist for Early Recognition and Treatment of Acute Illness and Injury critical care training program (2016‐2022).

Course type, location, and year^a^		Faculty members (n)	Learners (n)	Course duration (h)
Rochester course (in-person)^b^				
2016/Rochester		6	11	8
2017/Rochester		6	11	8
2018/Rochester		6	19	8
2019/Rochester		6	16	8
Subtotal		24	57	32
International course (in-person)^c^				
2019/New Delhi, India		3	40	8
2019/Zagreb, Croatia		3	28	8
2019/Ljubljana, Slovenia		5	61	8
2019/Guangzhou, China		5	93	8
2019/Hanoi, Vietnam		5	41	8
2019/Beijing, China		5	27	8
Subtotal		26	290	48
Online^d^				
2020/China		3	32	40
2021/Bosnia		3	107	40
2021/Montenegro		3	74	40
2022/Vietnam		3	69	40
Subtotal		12	282	160

a Study design: cross-sectional analysis; population: 629 learners/62 faculty members; locations: Rochester (US), India, Croatia, China, Slovenia, Vietnam, Bosnia, and Montenegro; timeframe: 2016‐2022

b Rochester course: in-person training conducted annually in Rochester.

c International course: in-person training conducted in various international locations.

d Online: courses delivered virtually in the specified years and regions.

**Figure 1.**
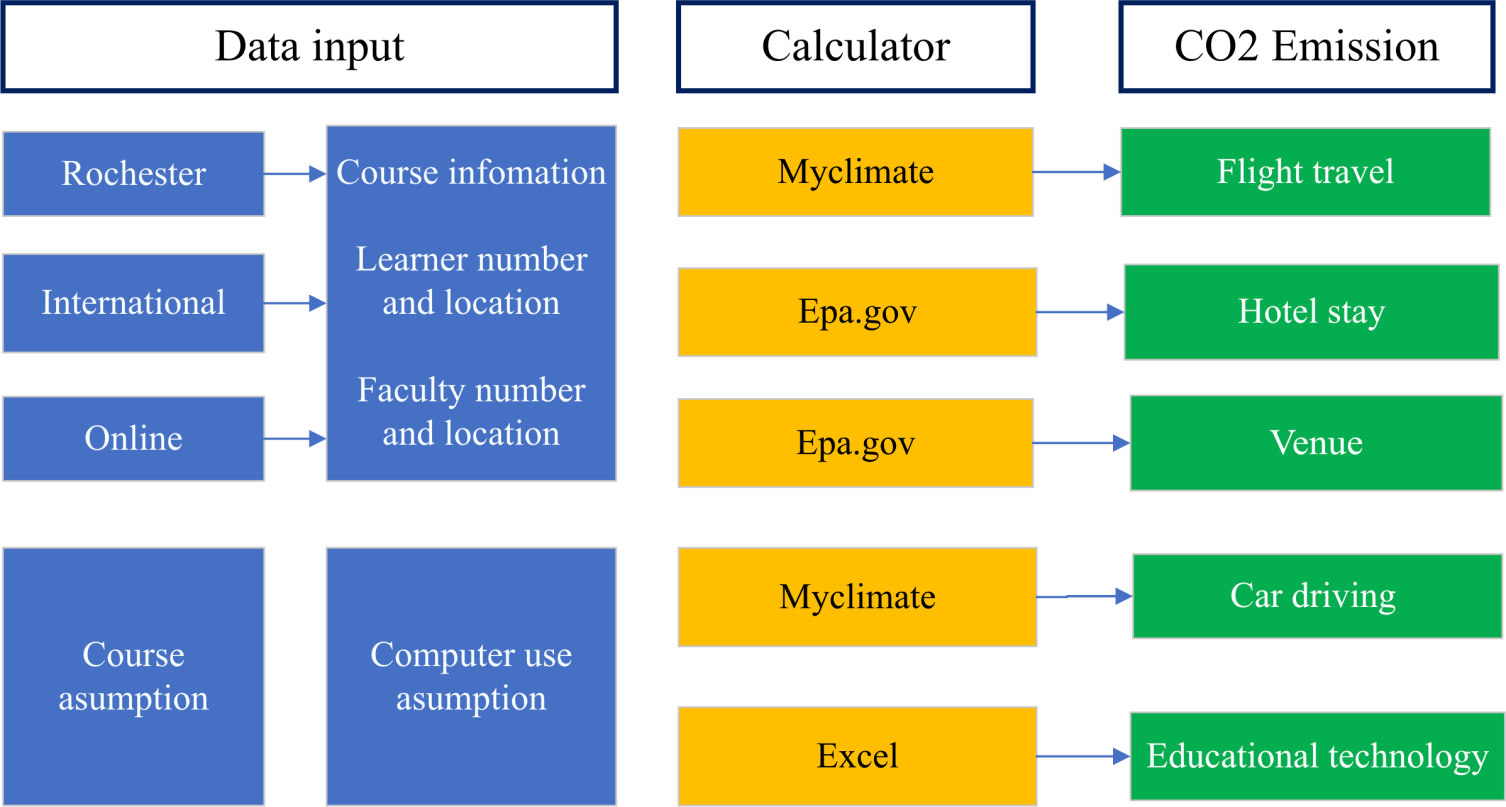
Carbon footprint calculation framework for the CERTAIN program using my climate calculator and EPA standards. carbon dioxide (CO_2_)

We performed a comprehensive analysis of the CERTAIN Course’s carbon footprint, including CO2e emission factors for travel, hotel, conference facility venues, local transportation, and educational technology (personal computers, network data transfer, servers, and organizer meetings) (Figure 1).

The assumption was used for the following categories:

1. Flight travel: We used the My Climate flight calculator [19], a publicly accessible internet-based tool, to determine direct and indirect CO_2_e emissions per airplane passenger for each specified flight distance. This calculation used the average fuel burn rates of common aircraft. We assumed that all passengers flew economy class and traveled from their respective capital cities to Rochester and back.
2. Hotel stay: To calculate emissions produced by hotel stays. We used energy consumption data from the Environmental Protection Agency's (EPA) resources, “Indirect Emissions from Events and Conferences” [20]. The accommodation provided fell under the “Midscale with food and beverage” category, and the event was hosted in the Midwest region. We assumed registrants to stay in the “Midscale” hotel hosting the conference for 2 nights.
3. Venue: To calculate venue-related emissions, we used emission factors sourced from Environmental Protection Agency (EPA) datasets, “Indirect Emissions from Events and Conferences” [19]. We assumed using 1500 square feet of meeting space at the venue.
4. Car driving: we used “My Climate” to calculate driving by car emissions [21]. Our method was grounded in defined assumptions: The car with a petrol fuel type, consuming 8 liters per 100 kilometers, emits 0.033 metric tons (33 kilograms) of CO_2_. Each attendee was assumed to make a round trip from their residence to the event location, a distance estimated at 5 kilometers separated from the hotel and the venue, with faculty members incurring an equivalent travel distance. Additionally, the transit from the airport to the hotel, a 20-kilometer journey, was anticipated to be undertaken twice by each participant.
5. Educational technology: We used methodologies adapted from Faber’s research [22,23] to calculate learners’ computer CO_2_e emissions from various components, including computer use, network data transfer, and electricity consumption associated with both personal devices and servers. We calculated the carbon footprint of the computers used by the participants and the organizers of the online conference.

In the Rochester courses and international courses, we assumed about one-fourth of learners used computers. We assumed that everyone used the same model of computer for 8 hours on the conference day and 1 hour for each course meeting. The faculty also attended an organizer meeting for 8 hours.

We calculated the emissions for the online course assuming each learner used the same model of computer. The course duration was 40 hours, and the organizer meeting was 1 hour. We also assumed that 2 faculty members attended the organizer meeting and used the same computer model as the learners.

### Data Collection

This study used a secure online database for data collection, gathering detailed information on faculty and learners of the CERTAIN course, including course duration and learner origins. We used Microsoft Excel to calculate flight emissions, hotel stay emissions, venue emissions, car driving emissions, and educational technology (computer use, network data transfer, and electricity consumption associated with both personal devices and servers).

### Statistical Analysis

Continuous variables are presented as mean (SD). Differences in average carbon dioxide equivalent (CO₂e) emissions (per course and per participant) across the 3 delivery formats—local in-person (Rochester, MN; n=4 courses, 81 participants), international in-person (n=6 courses, 316 participants), and online (n=4 courses, 294 participants)—were analyzed using one-way ANOVA in IBM SPSS Statistics (version 28.0; IBM Corp). Normality of data distribution was confirmed via the Shapiro-Wilk test (*P*>.05 for all groups), and homogeneity of variances was verified using Levene test (*P*>.10). Post-hoc pairwise comparisons were conducted with Tukey Honest Significant Difference test to adjust for multiple comparisons (*α*=.05).

### Ethical Considerations

The Mayo Clinic Institutional Review Board (IRB) acknowledges that based on the responses submitted for this new activity through the Mayo Clinic IRBe Human Subjects Research Wizard tool, and in accordance with the Code of Federal Regulations, 45 CFR 46.102, the above noted activity does not require IRB review.

## Results

### CERTAIN Program Course Modalities

#### Course Implementation and Participation

The CERTAIN program was delivered through three distinct modalities between 2016 and 2022, reaching a total of 629 learners across 98 faculty members.

#### Rochester Course (In-person)

The Rochester in-person course was conducted annually from 2016 to 2019. A total of 24 faculty members delivered the program to 57 learners over four years, with each course lasting 8 hours. Annual enrollment ranged from 11 to 19 participants, with the highest enrollment recorded in 2018 (n=19).

#### International Course (In-person)

Six international in-person courses were implemented across five countries in Asia and Europe. These courses engaged 26 faculty members and trained 290 learners, representing the largest participant cohort among the three modalities. Each course maintained the standard 8-hour duration. The highest enrollment was observed in Guangzhou, China (n=93), followed by Ljubljana, Slovenia (n=61). Course sizes varied considerably, ranging from 27 participants (Beijing, China) to 93 participants (Guangzhou, China).

#### Online Course

Four online courses were delivered between 2020 and 2022, primarily targeting participants in Asia and the Balkans. A total of 12 faculty members facilitated these courses for 282 learners. Unlike the in-person modalities, online courses were extended to 40 hours duration. The largest online cohort was recorded in Bosnia (2021) with 107 participants, while the smallest was in China (2020) with 32 participants (Table 1).

Calculated CO_2_e emissions for each of the 3-course offerings are summarized in Table 2 with detailed calculations in Multimedia Appendix 1. The local Rochester, MN course, involving a consistent faculty number and varying international learner enrollments, was responsible for a total of 210.8 tons of CO_2_e emissions. The results showed a year-on-year increase in emissions, from 22.4 tons CO_2_e in 2016 to a peak of 78.8 tons CO_2_e in 2018, with only a slight reduction to 65.7 tons CO_2_e in 2019. This difference was due to the variation in learners’ number. Course participation resulted in an average emission of 2.5 tons CO_2_e per participant and 52.7 tons CO_2_e per course (Table 2).

**Table 2.** Breakdown of carbon dioxide equivalent emissions by source across instructional modalities unit (metric tons).

Course type, location, and year^a^^b^				Carbon dioxide equivalent emissions by category (kg)^c^						Total/average emission per course (tons)	Average CO_2_ emission per person (tons)
				Flight travel	Hotel stays	Venue	Driving by car	Educational technology^d^	Total		
Rochester course (in-person)											
2016				21,500	398.2	64	178.2	229.3	22,369.7	22.4	1.3
2017				43,000	398.2	64	244.2	229.3	43,935.7	43.9	2.6
2018				77,400	687.8	64	402.6	243.9	78,798.3	78.8	3.2
2019				64,500	579.2	64	343.2	236.6	65,723	65.7	3
Total				206,400	2063.4	256	1168.2	939	210,826.6	210.8/52.7	2.5
International course (in-person)											
New Delhi				15,600	108.6	64	323.4	258.5	16,354.5	16.4	0.4
Zagreb				9000	108.6	64	244.2	236.6	9653.4	9.7	0.3
Ljubljana				14,500	181	64	501.6	309.7	15,556.3	15.6	0.2
Guangzhou				27,000	181	64	712.8	368.2	28,326	28.3	0.3
Ha Noi				28,500	181	64	369.6	273.1	29,387.7	29.4	0.6
Beijing				21,500	90.5	32	171.6	251.2	22,045.3	22	0.7
Total				116,100	850.7	352	2323.2	1697.3	121,323.2	121.4/20.2	0.4
Online											
2020, China				0	0	0	0	4170	4170	4.2	0.1
2021, Bosnia				0	0	0	0	6911.6	6911.6	6.9	0.1
2021, Montenegro				0	0	0	0	5705.3	5705.3	5.7	0.1
2022, Vietnam				0	0	0	0	5522.5	5522.5	5.5	0.1
Total				0	0	0	0	22,309.4	22,309.4	22.3/5.6	0.1

a Study design: cross-course comparison.

b Geographic scope: multinational; timeframe: 2016‐2022.

c Emissions: local courses (52.7 metric tons/course, 2.5 metric tons/per person), international courses (20.2 metric tons/course ,0.4 metric tons/per person), and online courses (5.6 metric tons/course, 0.1 metric tons/per person).

d Educational technology emissions include learner’s computers, network data transfer, server emissions, and faculty meetings.

The collective carbon emissions from the CERTAIN international courses in 2019 amounted to 121.3 metric ton of CO_2_e. Air travel was again identified as the primary source of emissions, accounting for over 93% in each case. Course participation resulted in an average emission of 0.4 tons CO2e per participant and 20.2 tons CO_2_e per course (Table 2).

CO_2_e emissions of online CERTAIN courses totaled 22.3 tons over the 3 years. Despite the scale of these courses—spanning multiple countries and involving a significant number of learners and faculty—the average CO_2_e emission per participant was only 0.1 tons CO_2_e, and per course was 5.6 tons CO_2_e emissions (Table 2).

Post-hoc analysis using Tukey HSD test indicated that the CO₂ emissions of the Rochester course were significantly higher than those of the international and online courses (*P*<.001). Additionally, the CO₂ emissions of the international course were significantly higher than those of the online course (*P*<.001) (Table 3).

**Table 3.** Statistical comparison of carbon dioxide equivalent emissions across instructional modalities (ANOVA).

Variables^a^^b^	In-person course ,Rochester	International course	Online course	*F* test (*df*)^c^	*P* value^c^	Post-hoc^d^
Average carbon dioxide equivalent emissions per course (tons);^e^ n, mean (SD)	4, 52.71 (25.5)	6, 18.55 (8.39)	4, 5.58 (1.2)	24.67 (2,11)	<.001	R^f^>I^g^> O^h^
Average carbon dioxide equivalent emissions per person^i^ (tons); n, mean (SD)	81, 1.30 (0.25)	316, 0.40 (0.15)	294, 0.10 (0.02)	18.45 (2,688)	<.001	R>I > O

a Study design: variance analysis.

b Locations: United States, India, and 3 other countries; timeframe: 2016-2022.

c One-way ANOVA results are reported with *F* test and *P* value.

d Post-hoc (Tukey honest significant difference): symbols indicate pairwise comparisons (“>” denotes significant differences). Post-hoc tests confirmed Rochester >International >Online.

e Average per course (kg): Significant differences were observed between course types (*F*_2,11_=24.67, *P*<.001).

f R: Rochester.

g I: international.

h O: online.

i Average CO₂ per person (tons): Significant differences were also found (*F*_2,688_=18.45, *P*<.001), with the same hierarchical pattern (Rochester>International>Online).

A comparison of the average emissions per course for each of the 3 CERTAIN program formats is summarized in Table 4. Rochester leads in CO_2_e emissions, both in total and per capita, significantly surpassing International and Online, which show much lower emissions in comparison. Notably, online shows the lowest emissions in comparison.

**Table 4.** Post-hoc analysis of carbon dioxide equivalent emission differences across instructional modalities using Tukey Honest Significant Difference test.

Comparison^a^^b^ variables	Emissions, mean difference (tons)	*P* value^c^
Average emissions per course		
Rochester versus international	3.42	<.001
Rochester versus online	4.71	<.001
International versus online	1.30	<.001
Average CO₂ per person		
Rochester versus international	0.90	.002
Rochester versus online	1.20	<.001
International versus online	0.30	.005

a Study design: multiple comparisons.

b Geographic scope: multinational; timeframe: 2016‐2022.

c Significant differences: local versus international (*P*.001), local versus online (*P*<.001), international versus online (*P*=.005).

## Discussion

### Principal Findings

Online education has been adopted by the medical field and has been growing after the COVID-19 pandemic [13]. Health care professionals are also aware of the impact of the health care industry on the environment [24]. Our analysis underscores the considerable environmental benefits of transitioning academic meetings and conferences to online platforms. In comparing the carbon footprints of CERTAIN courses delivered in Rochester, internationally, and online, we observed online delivery resulted in 98% and 61% lower GHG impact compared to the in-person Rochester and international courses, respectively, due to reduced travel requirements. Our findings are consistent with a growing body of evidence suggesting that increasing the use of online platforms for academic meetings and conferences offers an effective strategy to lessen the environmental impact of these activities [25]. Roy et al [26] demonstrated that distance learning in higher education consumes 87% less energy and produces 85% lower CO_2_e emissions than traditional full-time campus-based courses. Similarly, part-time campus courses are also more efficient, reducing energy and CO_2_e emissions by 65% and 61%, respectively. The primary contributing factors to these reductions are decreased student travel, reduced energy consumption for student housing, and more efficient campus site use [26].

Transportation already accounts for up to 26% of global CO_2_e emissions and is one of the few industrial sectors where emissions continue to increase [27]. Traveling by airplane has been considered the main factor contributing to the high carbon footprint of medical congresses around the globe [28]. Climate experts warn that the Paris Agreement’s target of limiting global warming to 1.5°C by 2050 requires drastic cuts in greenhouse gas emissions, with target individual emissions of 2.5, 1.4, and 0.7 tons of CO_2_e per year by 2030, 2040, and 2050, respectively [29]. The average American emits 14.9 tons in 2022, and the global average is 4.6 tons of CO_2_e [2,30]. For example, a single conference trip can account for as much as 7% of an average individual’s total annual CO2e emissions [31]. Multiple authors have demonstrated a dramatic improvement in CO2e leveraging virtual continuing medical education delivery and eliminating these costly travel requirements [13,23,32-35]. Adopting online and hybrid educational strategies aligns the medical field with sustainability goals, setting a precedent for environmentally responsible practices [36].

Online platforms also ensure that high-quality and equitable learning opportunities are accessible to a global audience, regardless of geographical location or economic status [37]. This accessibility improves the delivery of high-quality evidence-based care to patients, as health care professionals who engage in simulation courses would be able to apply their learned skills directly to their clinical practice [38-40]. Additionally, it fosters international collaboration and broadens global clinical and research opportunities by sponsoring exchanges of research scholars and intensivists among institutions [37,38,41].

However, the available platforms of online classes do not provide a classroom-like feel, thereby creating hindrances in students’ learning process and diminishing the quality of education [42]. While many have raised valid concerns about the quality of online compared to in-person educational experiences, especially on factors like poor Internet access, student engagement, interaction, and access to resources [43], there is also a lot of promise in the initial findings. Research by Almendingen et al and Clark et al [44,45] found that students adapted well to online learning during the COVID-19 pandemic, showing improved academic performance. Specifically, Clark et al [45] reported that students who accessed higher-quality, externally recorded lessons significantly outperformed peers who used their own school’s recordings, showing the benefits of quality educational resources. Gonzalez et al [46] found that a large cohort of students from the Universidad Autónoma de Madrid developed more consistent learning habits using an online learning model during the COVID-19 pandemic, which positively influenced academic performance [46]. This finding is reinforced by observations in India, where online tests and open-book exams have also been correlated with better student scores [46]. In our experience, transitioning to a digital platform for medical education posed challenges but achieved similar rates of learner satisfaction [17].

Online learning also offers a solution by eliminating the need to travel, thus preventing the risk of acute sleep deprivation and its effects on performance and memory processing [47,48]. This enables participants to engage fully and efficiently in their educational activities with greater flexibility by joining live or watching video recordings at the time of their convenience.

While the benefits of international online learning are evident, its limitations, including technological and language barriers, must also be acknowledged [40]. However, in-person, hands-on experience is still necessary in many aspects of medical education, especially in procedural training [49]. Online education not only aligns with worldwide efforts to mitigate climate change and global warming but also offers a pathway to enhance inclusive, flexible, and accessible education. This suggests a hybrid model may be a promising future direction, integrating the strengths of both online and in-person learning platforms [50] to create an optimal balance, particularly for practical skill acquisition. Additionally, it suggests the need for further research into the environmental impacts of incorporating advanced technologies like virtual reality into medical training. Finally, our findings underscore the need for broader awareness and analyses using these methods on the long-term effects of online medical education on global sustainability and, to better understand its overall impact [51].

### Study Limitations

There are some limitations of our study. Emission calculators require a variety of different assumptions, which can result in variability in the resulting estimates of carbon emissions and their environmental impact. Our approach might not fully capture actual flight emissions due to missing variables like flight class or route changes. Car travel calculations, based on straight-line distances and gasoline-powered vehicles, may not reflect actual fuel consumption. We also were not able to include emissions from nonparticipant companion travel and ancillary activities outside of conference participation, which resulted in an underestimation of the total emissions impact. Additionally, we found it was impossible to account for all online activity–related emissions and could not capture such common sources as search engine queries, monitor usage, desk lamp usage, and website visits. The study also assumed uniform computer usage among participants, oversimplifying diverse technology use and energy consumption. In general, however, these unaccounted emissions were considered minor relative to the overall footprint of online learning. Our study is a retrospective analysis of the previous course. There is a great need to develop an online calculator like in the airline industry as a planning tool for medical education activities [52].

### Conclusions

Online medical education offers significant environmental benefits and increased global availability over traditional in-person methods. With high course ratings and comparable learner satisfaction, our findings join a growing body of literature suggesting a strategic shift toward digital or hybrid learning platforms. This transition is seen as key to enhancing sustainability, accessibility, and global reach in medical education, aligning with global environmental goals and evolving educational needs.

## Supplementary material

10.2196/63524 Multimedia Appendix 1 Characteristics of three instructional modalities in the Checklist for Early Recognition and Treatment of Acute Illness and Injury critical care training program

## Abbreviations

CERTAIN: Checklist for Early Recognition and Treatment of Acute Illness and Injury
CO_2_e: carbon dioxide equivalent
GHG: greenhouse gas
ICU: intensive care unit
